# ﻿Two new species of the *Phaoniaboleticola*-group (Diptera, Muscidae, Phaoniinae) from China

**DOI:** 10.3897/zookeys.1168.97845

**Published:** 2023-07-03

**Authors:** Zhongyan Zhou, Lianmeng Wei

**Affiliations:** 1 Anshun University, Anshun, Guizhou, 561000 China Anshun University Anshun China; 2 Centre for Disease Prevention and Control of Anshun City, Anshun, Guizhou, 561000 China Centre for Disease Prevention and Control of Anshun City Anshun China

**Keywords:** *boleticola*-group, key, *Phaonia*, southwestern China, taxonomy

## Abstract

Two new species of *Phaonia* are described: *Phaoniaagitata* Zhou & Wei, **sp. nov.** and *Phaonianujiangensis* Zhou & Wei, **sp. nov.**, which were collected from Guizhou and Yunnan provinces of southwestern China and are assigned to the *boleticola*-group. A key to the species of this group is provided. The type specimens are deposited in the Wei Lianmeng Model Worker Innovation Studio, Anshun, Guizhou, China (WLMWISAGC).

## ﻿Introduction

The genus *Phaonia* Robineau-Desvoidy, 1830 with the important synapomorphy – 1 strong *pd* (calcar) present at apical 1/4 of hind tibia – is one of the largest genera of Muscidae, mainly distributed in Palaearctic and Oriental regions (Sorokina 2015). So far, some 806 species are known from the world and nearly half of them are described from China (Xue et al. 2014). The first author to identify groups in *Phaonia* was W. Hennig who established 18 groups of species in his Palaearctic monograph, including the *boleticola* species-group with four species (Hennig 1963). To date, 39 species groups are known worldwide, which are based only on external characters (Ma et al. 2002; Xue et al. 2008). However, there is at present a lack of reliable phylogenetic evidence, and so there may be a need to erect additional species groups. In the *boleticola* species-group, 10 species have been recorded worldwide, all distributed in the Palearctic region except for *hainanensis* which occurs in the Oriental region, and seven species have been reported from China (Xue et al. 2008). During the identification of specimens collected from Guizhou, Sichuan and Yunnan provinces in Southwest China, two species of the *boleticola*-group from Guizhou and Yunnan were identified as new to science.

This paper is a result of the study of specimens deposited in the Wei Lianmeng Model Worker Innovation Studio, Anshun, Guizhou, China (WLMWISAGC) and is part of on-going studies on the genus *Phaonia* from Guizhou, Sichuan and Yunnan provinces of southwestern China. A key to all seven species of the *P.boleticola* species-group from China is provided.

## ﻿Materials and methods

Material for this study came from specimens collected in southwestern China between 1993 and 2013. The terminalia were prepared as follows: the last abdominal segment of relaxed specimens was carefully removed with microscissors and was then put into 10% KOH solution. It was either boiled for 5–10 min or kept for 24 h at room temperature until all the parts became clear. The dissected male terminalia were placed in glycerine, balanced on some cotton wool, and drawn using a camera lucida attached to a compound microscope. Morphological terms generally follow McAlpine (1981), but Cumming and Wood (2017), Stuckenberg (1999) and Shinonaga (2003) were used for some structures. Body length is measured from the base of the antenna to the tip of the abdomen. The following abbreviations are used: ***a***–anterior seta, acroph–acrophallus, *acr*–acrostichal seta, ***ad***–anterodorsal seta, **aed-apod**–aedeagal apodeme, **aed**–aedeagus, **av**–anteroventral seta, **cerc**–cercus, ***dc***–dorsocentral seta, ***c***–costal vein of wing, **dm-cu**–discal medial-cubital cross-vein of wing, **ej-apod**–ejaculatory apodeme, **epand**–epandrium, **epiph**–epiphallus, **M**–medial vein of wing, **m-m**–inter-medial cross-vein of wing, ***p***–posterior seta, ***pd***–posterodorsal seta, **pgt**–postgonite, ***pra***–prealar seta, **pregt**–pregonite, ***pv***–posteroventral seta, **R_4+5_**–branch of radius of wing, **r-m**–radial-medial cross-vein of wing, **st 1**–sternite 1, **st 5**–sternite 5, **tg**–tergite, **tg 1+2**–1^st^ and 2^nd^ syntergite, **tg 3, 4, 5**–tergites 3, 4, 5.

## ﻿Taxonomic account

### ﻿ Phaonia boleticola

Taxon classificationAnimaliaDipteraMuscidae

species-group

344A09BE-A84D-55E8-9FC3-875031FE51A8

Phaonia boleticola species-group Ma et al., 2002: 243–246; Xue et al. 2008: 42 (1): 155–171.

#### Diagnosis.

Members of the *P.boleticola* species-group have the following combination of characters: frons narrow, arista plumose, presutural *acr* 0, *pra* undeveloped, prosternum bare, scutellum and abdomen dark, mid tibia with a row of *p* or *pv* only, hind tibia without apical *pv*.

Except as noted above, the authors find the male terminalia of the *P.boleticola* species-group to have the following characters: viewed posteriorly, cerci usually vest-like, fused proximally on upper 1/2 to 3/5, or even nearly completely fused.

#### Remarks.

Both *P.xuei* Wang & Xu, 1998 and *P.fani* Ma & Wang, 1992 (only females known) are different from other members of the *P.boleticola* species-group in the following features: abdomen not entirely black, tg 1+2 and most of tg 3 pale yellow; tg 4 and 5 each with hind marginal band yellow; male cercus of *P.xuei* rounded, which is very different from that of other species of the *P.boleticola* species-group. Therefore, these two species probably do not belong to the *P.boleticola* species-group or the definition of the *P.boleticola* species-group needs to be studied further and modified.

#### Included species.

Nine species including the two new species described herein: *P.agitata* sp. nov., *P.cyclosternita* Xue, 1996, *P.fani* Ma & Wang, 1992, *P.hainanensis* Xue, 2008, *P.nigripennis* Ma & Cui, 1992, *P.nujiangensis* sp. nov., *P.suscepta* Xue, 1996, *P.suspiciosa* (Stein, 1907) and *P.xuei* Wang & Xu, 1998.

#### Affinities.

The *P.boleticola*-group is close to the *P.nymphaearum*-group but can be differentiated from the latter by the plumose arista.

#### Distribution.

China (Palearctic and Oriental regions). *Phaoniaboleticola* (Rondani, 1866) is distributed through Central Europe to Middle Asia.

### ﻿ Phaonia agitata

Taxon classificationAnimaliaDipteraMuscidae

Zhou & Wei sp. nov.

C733D5DA-7C61-5347-B968-68F005D133FA

https://zoobank.org/4A0247D4-4190-4A92-8DD9-7823D065B7BC

Figs 1–5 , 11–14

#### Types.

***Holotype*. China**: “Guizhou, Fanjing Mountain, 1800 m, 27°55’N, 108°41’E, 22.VI.1994, LM Wei”, (WLMWISAGC, 1 male). ***Paratypes*.** Same data as holotype (WLMWISAGC, 2 males).

#### Diagnosis.

Eye bare, antenna brownish-yellow; *dc* 2+3, katepimeron setulose, both anterior and posterior spiracles brown; legs dark brown, with apex of femora and trochanters brown, tibiae yellow-brown and tarsus black, somewhat brownish, mid tibia with 2 *p*, hind tibia with 3 *av*; male terminalia: in posterior view, cerci with distal outer angle dully rounded; in profile, surstylus short, with apex widened; st5 (Fig. 3) oval, apical angle of lateral lobe pointed and curved inwards. Aedeagus with acrophallus triangular, membranous. Ejaculatory apodeme diamond-shaped.

**Figures 1–5.**
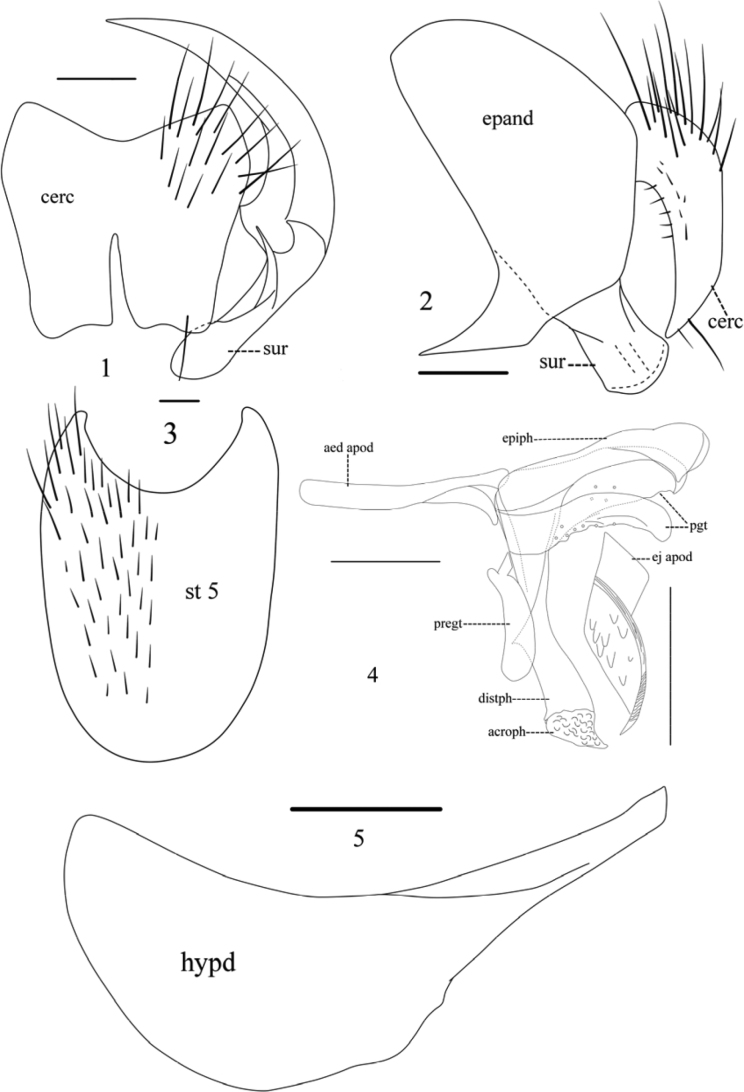
*Phaoniaagitata* Zhou & Wei, sp. nov. **1, 2** cercus in posterior and lateral views **3** sternite 5 in ventral view **4** terminalia, lateral view **5** hypandrium, lateral view. Scale bar: 0.1 mm.

#### Description.

**Male.** Bright black species, thorax grey-brown pollinose, scutellum dark. Viewed posteriorly, scutum with 4 longitudinal dark vittae. Wing darkish brown. Legs dark brown, femoral tips and trochanters brown, tibiae yellow-brown and tarsus black, somewhat brownish.

***Head*** with eye bare, anterior inner facets distinctly enlarged. Frons somewhat wider than the space between outer margin of two posterior ocelli. Frontal vitta black, devoid of pruinosity, about as wide as facial ridge. Fronto-orbital plate dark, densely silver-grey pollinose. Frontal setae 5 pairs, mixed with individual setulae, uppermost one as weak as setulae, the distance from this to anterior ocellus about 1.5 times the space between outer margin of two posterior ocelli. Lunule darkish tawny-yellowish, silver-grey pollinose. Antenna brownish-yellow, postpedicel brownish apically, scape and pedicel brown; postpedicel about 2.3 times as long as pedicel or about 3.3 times as long as wide. Arista brownish-yellow, thickened basally, with long plumosity, the longest rays about as long as width of postpedicel. Face and facial ridge mainly black, more or less yellowish, brown-grey pollinose. Lower facial margin not protruding in profile. Genal groove dark red dark, almost without pruinosity. Gena black, brown-grey pollinose, about 0.18 times of eye height or equal to 1.5 times width of postpedicel, its anterior margin with a row of upcurved setae. Palpus rod-like, black, longer than haustellum. Prementum stout, brown, thinly grey pollinose. Labellum developed, dark red. Occiput black, grey pollinose, with dark setae.

***Thorax*.***Acr* 0+1. *Dc* 2+3. *Pra* short, at most as long as posterior notopleural seta. Anterior postpronotal seta developed, longer than postpronotal seta. Scutellum bare laterally and ventrally. Anterior and posterior spiracles brown. Katepisternal setae 1:2. Katepimeron setulose. Prosternum, notopleuron and meron bare.

***Wing*** with veins brown to dark brown. Tegula and basicosta black. Costal spine undeveloped, clearly shorter than cross-vein r-m. *C* setulose ventrally, but 5^th^ section bare. Radial node bare dorsally and ventrally. Cross-veins r-m and m-m without much clouding and the latter curved. Distal part of R_4+5_ curved up and M straight and both of them gradually diverging towards apex. Calypters yellow, lower one narrow, tongue-shaped, with distal margin round, inner margin separated from scutellar margin. Haltere reddish-yellow.

***Legs*.** Fore tibia with a median *p.* Mid femur with a row of short and dense *a* on basal half, a row of setula-like *av* and *pv*, without *ad*; apically with 2 *ad* and 3 *pd.* Mid tibia with 2 *p.* Hind femur with a row of developed *ad*, 6 *av* at apex and a row of setulae-like *pv*; apically with 2 *ad*, 1–2 *pd* and 1 *p.* Hind tibia with 3 *av*, proximal one developed, 1 strong *pd* (calcar) present at apical 1/4, without apical *pv*.

***Abdomen*** oval, of the same colour as thorax. Tg1+2 with a dark tongue-shaped median patch. Tg3 with long and narrow dark cone-shaped median vitta. Tg4 and 5 each with a very narrow median vitta. Tg6 bare, dark brown. St1 bare. Male terminalia: Viewed posteriorly (Fig. 1): Cerci vest-like, fused on upper half of approximated portion and clearly invaginated at apex, distal outer angle bluntly rounded, inner angle with an apical seta. Surstylus spherical swollen apically. Viewed laterally (Fig. 2): Cercus inwardly curved conically. Surstylus strip-like, not elongated, narrow medially, with apex arched. St5 (Fig. 3) oval, apical angle of lateral lobe pointed and curved inwardly. Hypandrium (Fig. 5) spoon-shaped, its ventral margin deep incurvate, curved basoposteriorly. Genital tergite (Fig. 4) with pregonite slender, slightly curved, rounded apically. A pair of asymmetric postgonites, rather developed, the left one rounded apically, apparently smaller than the right one; the right postgonite hooked, pointed apically. Distiphallus elongated, the base infundibular, well sclerotized, widened toward apex. Acrophallus triangular, membranous. Aedeagal apodeme slender, bifurcate basally. Epiphallus developed strap-like, with apex expanded globe-like. Ejaculatory apodeme (as in Fig. 4) diamond-shaped with a sclerotized band medially and a papillary bulge on the surface.

**Female**. Unknown.

#### Measurements.

**Male**. Body length 6.9–7.0 mm.

#### Etymology.

The Latin *agitatus* means “agitated”, which refers to the excitement that the authors experienced when involved in fieldwork activity.

#### Distribution.

China (Fanjing Mountain, Guizhou).

#### Remarks.

The new species resembles *P.hainanensis* Xue et al. and these two species belong to the *P.boleticola*-group because they share the following characters: legs partly yellow, *dc* 2+3, meron bare, abdomen with a black median vitta and without a shining patch. It can be separated from the latter by the following combination of features: frons somewhat narrower than 2.5 times anterior ocellus, antenna brownish-yellow; male terminalia: in posterior view, cercus is wider, with distal outer angle dully rounded; in profile, surstylus short, with apex widened; st5 with apical angle of lateral lobe never expanded outwards when viewed ventrally. In *P.hainanensis*, the antenna is yellow; the cercus is narrower, with the distal outer angle acute; surstylus elongated, with apex narrow; st5 with apical outer angle strongly expanded outwards.

This new species is also similar to *P.nujiangensis* sp. nov. but can be separated from the latter as follows: Frons somewhat wider than the space between outer margins of posterior ocelli; katepimeron setulose; both anterior and posterior spiracles brown; legs dark brown, tibiae yellow-brown; mid tibia with 2 *p*, hind tibia with 3 *av.* Male terminalia: Cercus as long as wide. Surstylus spherical, swollen apically, shorter in profile. St5 (Fig. 3) oval, apical angle of lateral lobe curved inwards. Aedeagus with acrophallus triangular, membranous (Fig. 4).

### ﻿ Phaonia nujiangensis

Taxon classificationAnimaliaDipteraMuscidae

Zhou & Wei sp. nov.

69504D23-1167-548C-A246-69627FDBC1A9

https://zoobank.org/32AFD009-CAA6-4903-8C5D-D13B0A641EC6

Figs 6–10 , 15–17

#### Types.

***Holotype*. China**: “Gaoligongshan, Nujiang River side, 580 m, Yunnan, 24°49’51.75”N, 98°46’12.00”E, 2.X.2011, LM Wei, TH Shou, Y Gu, P Jiang and WP Cao” (WLMWISAGC, 1 male). ***Paratype*. China**: Same data as holotype (WLMWI SAGC, 1 male).

#### Diagnosis.

Eye bare, frons black, somewhat narrower than the space between outer margin of two posterior ocelli; antenna brownish-yellow, gena black-red, grey pollinose, about 0.1 times eye height or narrower than width of postpedicel; *dc* 2+3, both anterior and posterior spiracles pale yellow, katepimeron bare; legs yellow, fore femur brownish-black, mid and hind femora each with apex somewhat darkened dorsally; tarsus black, somewhat brownish, mid tibia with 3 *p*, hind tibia with 2*av.* Male terminalia: Viewed posteriorly: Cerci nearly completely fused in approximated portion. Apex of surstylus square-shaped and swollen. Viewed laterally: Cercus nearly hooked, the hind margin flexed at a right-angle. Surstylus strap-like, base strongly and apex slightly widened, apex bluntly rounded and hind margin curved posteriorly. St5 almost jar-shaped, apical angle of lateral lobe narrow, not curved inwards. Aedeagus with a small oval membranous area apically; acrophallus tail-shaped, membranous. Ejaculatory apodeme long, narrowly triangular.

#### Description.

**Male.** Black species. Thorax appearing dark red in ground-colour, thickly grey-brown pollinose. Wing brownish-yellow. Legs yellow, fore femur brownish-black, mid and hind femora somewhat darkish dorsally at apex; tarsus black, somewhat brownish. Abdomen elongate conical, reddish-brown, thinly grey-brown pollinose.

***Head*.** Eye bare, anterior inner facets distinctly enlarged. Frons black, somewhat narrower than the space between outer margin of two posterior ocelli. Frontal vitta devoid of pruinosity, completely disappeared at narrowest point. Fronto-orbital plate densely silver-grey pollinose. Frontal setae 6 pairs, upper two as weak as setulae, distributed on more than anterior half. Lunule darkish red-brown, silver-grey pollinose. Antenna brownish-yellow, postpedicel dark brownish apically, scape and pedicel darkish red-brown; postpedicel about 2.4 times as long as pedicel and also about 2.4 times long as wide. Arista darkish brown, thickened basally, long plumose, the longest rays as long as width of postpedicel. Facial ridge and median area black, silver-grey pollinose, the former less than half as wide as antennal width. Face bright black, thinly grey pollinose; facial ridge black but bright black along upper margin along a narrow strip, which is also thinly grey pollinose. Lower facial margin not protruding in profile. Gena black-red, grey pollinose, about 0.1 times eye height or narrower than postpedicel width, its anterior margin with a row of upcurved setae. Palpus rod-like, brownish-black, longer than haustellum. Prementum stout, dark brown, thinly grey pollinose. Labellum developed, dark yellow. Occiput black, grey pollinose, with dark setae.

***Thorax*** black, appearing dark red in ground-colour, thickly grey-brown pollinose. Viewed posteriorly, scutum with 4 longitudinal dark vittae, the inner pair obviously thin and stripe-like. Scutellum darkish red-brown, somewhat dark red in ground-colour, with hardly any pruinosity. *Acr* 0+1, as weak as setula. *Dc* 2+3. *Pra* short, about half as long as posterior notopleural seta. Anterior postpronotal seta developed, longer than postpronotal seta. Scutellum bare laterally and ventrally. Both anterior and posterior spiracles pale yellow. Katepisternal setae 1:2. Prosternum, notopleuron, meron and katepimeron bare.

***Wing*** brownish-yellow, veins yellow to dark brown. Tegula blackish-brown, basicosta brown. Costal spine developed, obviously longer than cross-vein r-m. *C* setulose ventrally on 1^st^ to 3^rd^ sections and basal half of 4^th^ sections. Radial node bare dorsally and ventrally. Cross-veins r-m and m-ms lightly clouded and the latter curved. Distal part of R_4+5_ and M straight, both somewhat diverging towards apex but parallel at apex. Calypters yellow, the lower one narrow, tongue-shaped, with distal margin round and inner margin separated from scutellar margin. Haltere yellow.

***Legs*** yellow, fore femur brownish-black, mid and hind femora somewhat darkish dorsally at apex; tarsus black, somewhat brownish. Fore tibia with a median developed *p.* Mid femur with a row of short and dense *a* on basal half, with 3 spine-like *av*, very short, without *pv*; apically with 2 *ad* and 3 *pd.* Mid tibia with 3 *p*, developed. Hind femur with a row of developed *ad*, 2 *av* at apex, proximal one developed, apically with 2 *d*, 1 *pd.* Hind tibia with 2 *av*, proximal one developed, 1 strong *pd* (calcar) present at apical 1/4, without apical *pv*.

**Figures 6–10.**
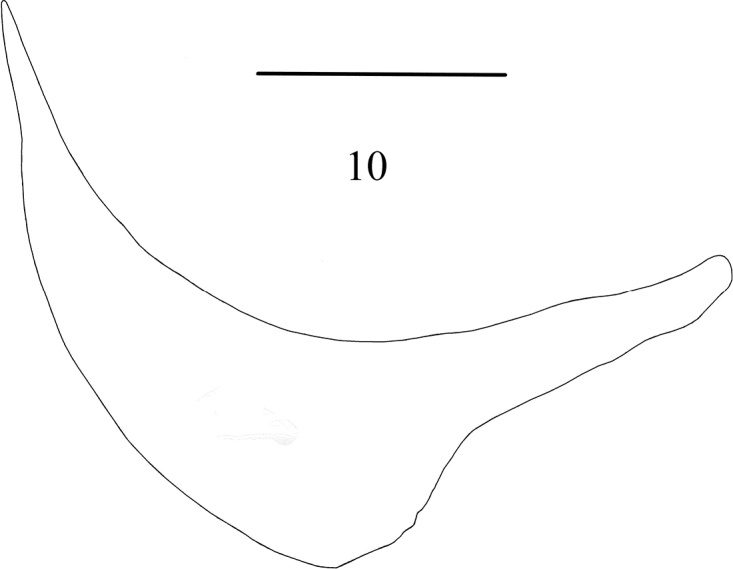
*Phaonianujiangensis* Zhou & Wei, sp. nov. **6, 7** cercus in posterior and lateral views **8** sternite 5 in ventral view **9** terminalia, lateral view **10** hypandrium, lateral view. Scale bar: 0.1 mm.

***Abdomen*** elongate conical, reddish-brown, thinly grey-brown pollinose. Tg3-5 with a dark median vitta, that on Tg5 very narrow and blurred at edges. Tg5 dark red-brown apically. St1 bare. Male terminalia: Viewed posteriorly (Fig. 6): Cerci vest-like, nearly completely fused in approximated portion and obviously invaginated at apex, apical outer angle bluntly rounded; inner angle with an apical seta. Apex of surstylus square-shaped and swollen. Viewed laterally (Fig. 7): Cercus nearly hooked, the hind margin flexed at a right-angle. Surstylus strap-like, moderately elongated, base strongly and apex slightly widened, apex bluntly rounded and hind margin curved posteriorly. St5 (Fig. 8) almost jar-shaped, apical angle of lateral lobe narrow, not curved inwards. Hypandrium (Fig. 10) meniscate, with deep median incision posteriorly. Genital tergite (Fig. 9): Pregonite slender, flexed from the middle, basal half curved cone-like, apical half strap-shaped, rounded apically. A pair of asymmetric postgonites, rather developed; the left one apparently smaller than the right one, base trapezoid and end curved, with 3 ventral setae medially; the right one wide strap-shaped, straight, rounded apically, with 4 ventral setae near the middle and 2 median setae. Distiphallus elongated, the base strap-shaped, well sclerotized, with a small oval membranous area apically. Acrophallus tail-shaped, membranous. Aedeagal apodeme slender, sclerotized apex expanded like a fan and wrapped round with a membranous plate. Epiphallus developed, rod-shaped, with apex expanded like a fan. Ejaculatory apodeme (as in Fig. 9) long, narrowly triangular.

**Female**. Unknown.

#### Measurements.

**Male.** Body length 4.9–5.0 mm.

#### Etymology.

The specific name refers to the type locality.

#### Distribution.

China, Yunnan (Nujiang River margin, Gaoligongshan).

#### Remarks.

The new species resembles *P.hainanensis* Xue et al., but this species differs from the new species by the yellow antenna; cerci narrower, with distal outer angle acute; surstylus elongated, with apex narrow; St5 with apical angle of lateral lobe not curved inwards. Furthermore, the new species is similar to *P.agitata* sp. nov. but can be separated from the latter by the following characters: frons somewhat narrower than the space between outer margin of two posterior ocelli; katepimeron bare; both anterior and posterior spiracles pale yellow; legs yellow, fore femur brownish-black; and male terminalia has different morphological characteristics from that of *P.hainanensis*.

**Figures 11–17.**
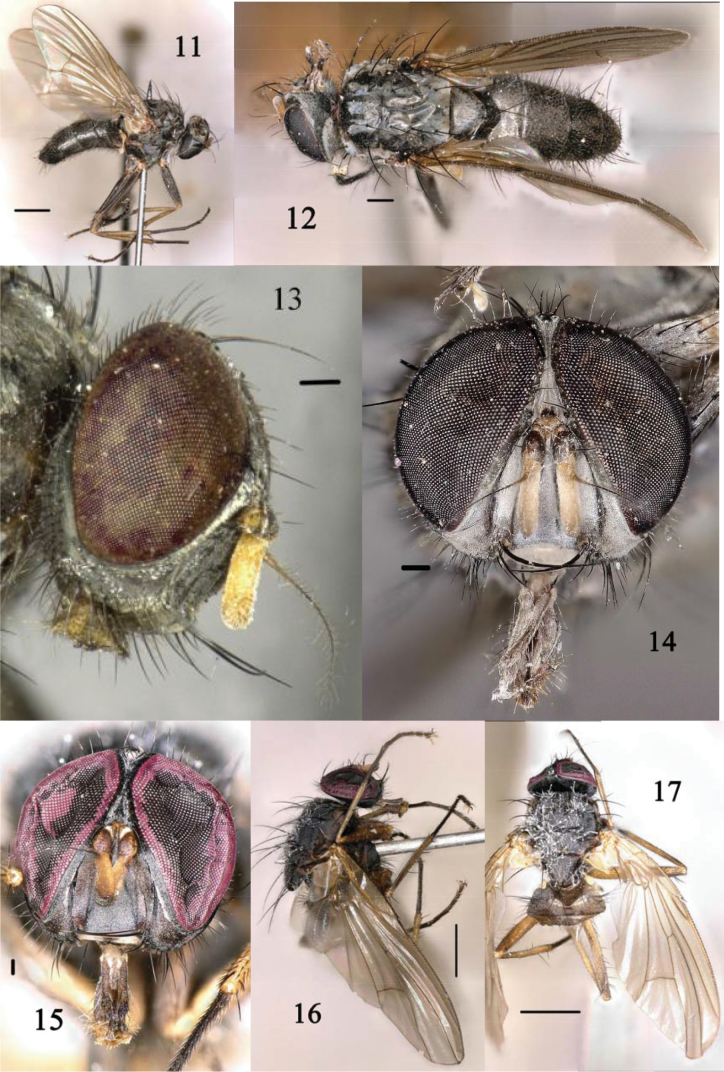
*Phaoniaagitata* Zhou & Wei, sp. nov. (♂) (**11–14)***Phaonianujiangensis* Zhou & Wei, sp. nov. (♂) (**15–17**) **11, 16** habitus in lateral view **13** head in lateral view **14, 15** head in anterior view **12, 17** thorax and wing in dorsal view. Scale bar: 0.2 mm.

### ﻿Key to species of the *Phaoniaboleticola*-group from China (males)

Modified from Xue et al. 2008.

**Table:** 

1	Legs entirely black. Meron setulose	**2**
–	Legs partly brown or yellow at least. Meron setulose or bare	**3**
2	Mid tibia with 1 *ad.* Wing base dark brown. Basicosta black	***P.nigripennis***
–	Mid tibia without *ad.* Wing hyaline or slightly brown. Basicosta dark brown	***P.cyclosternita***
3	Postsutural *dc* 4. Frons wider than 1.5 times the space between external margins of posterior ocelli	***P.fani***
–	Postsutural *dc* 3. Frons at most 1.5 times the space between external margins of posterior ocelli	**4**
4	Frons about 1.5 times the space between external margins of posterior ocelli	**5**
–	Frons at most somewhat wider than the space between external margins of posterior celli	**8**
5	Meron setulose. Eye sparsely covered with short cilia	***P.suspiciosa***
–	Meron bare. Eye bare, covered at most with a few short cilia	**6**
6	*Pra* absent. Antenna with postpedicel yellow or not yellow	**7**
–	*Pra* present, about half length of posterior postpronotal seta. Antenna with postpedicel yellow	***P.hainanensis***
7	Mid tibia with 1 *ad.* Palpus yellow. Antenna with base of scape and pedicel yellowish-brown. The area of cross-veins r-m and dm-cu unclouded. Calypters white	***P.xuei***
–	Mid tibia without *ad.* Palpus blackish-brown. Antenna entirely black. The area of cross-veins r-m and dm-cu unclouded. Calypter canary-yellow	***P.suscepta***
8	Frons somewhat wider than the space between external margins of posterior ocelli. Katepimeron setulose. Both anterior and posterior spiracles brown. Legs dark brown, with apex of femora and trochanters brown, tibiae yellow-brown and tarsus black, somewhat brownish. Mid tibia with 2 *p.* Hind tibia with 3 *av*	***P.agitata* sp. nov.**
–	Frons somewhat narrower than the space between outer margins of posterior ocelli. Katepimeron bare. Both anterior and posterior spiracles pale yellow. Legs yellow, fore femur brownish-black, mid and hind femora somewhat darkish dorsally at tips; tarsus black, somewhat brownish. Mid tibia with 3 *p.* Hind tibia with 2 *av*	***P.nujiangensis* sp. nov.**

## Supplementary Material

XML Treatment for Phaonia boleticola

XML Treatment for Phaonia agitata

XML Treatment for Phaonia nujiangensis

## ﻿Additional information

### Author ORCIDs

Zhongyan Zhou https://orcid.org/0000-0002-9477-2604

Lianmeng Wei https://orcid.org/0000-0002-2412-8119

### Data availability

All of the data that support the findings of this study are available in the main text or Supplementary Information.
